# ﻿First DNA barcodes and morphometric analysis of Singaporean Tetrigidae (Orthoptera, Caelifera) support color variations as intraspecific polymorphism through integrative taxonomy

**DOI:** 10.3897/zookeys.1257.163020

**Published:** 2025-10-30

**Authors:** Ivan Neo, Rebecca Ker Loh, Tricia J. Y. Cho, Nalini Puniamoorthy, Darren C. J. Yeo, Ming Kai Tan

**Affiliations:** 1 Department of Biological Sciences, National University of Singapore, 16 Science Drive 4, Singapore 117558, Singapore National University of Singapore Singapore Singapore; 2 Lee Kong Chian Natural History Museum, Faculty of Science, National University of Singapore, 2 Conservatory Drive, Singapore 117377, Singapore National University of Singapore Singapore Singapore

**Keywords:** *COI*, molecular analysis, objective clustering, phylogenetics, pygmy grasshoppers, *12S*

## Abstract

Integrative taxonomy refers to the practice of incorporating multiple lines of evidence, including morphology and genetics, to delineate species. While both morphology and genetic markers, such as the *Cytochrome c oxidase subunit I* (*COI*), have been widely applied in insect taxonomy, their use remains limited in the taxonomic studies of pygmy grasshoppers (Orthoptera: Tetrigidae). Currently, DNA barcodes for tetrigids, especially from Southeast Asia, remain scarce. This limitation is especially pressing given the extensive color variation documented in pygmy grasshoppers, which has rarely been systematically tested to determine whether such variation represents intraspecific polymorphisms or species differences. Historically, color polymorphism has even led to erroneous species delimitation. In this study, we examined taxa from Singapore previously reported to exhibit color variation and applied an integrative taxonomic framework to investigate whether these morphs represent intraspecific polymorphism or species-level differences. Our analyses combined morphometrics of 22 characters with principal component analyses, and DNA barcoding using short gene fragments of *COI* and small *ribosomal RNA subunit III* (*12S*). We focused on four taxa: two morphospecies of *Coptotettix*, *Loxilobus insidiosus* (Bolívar, 1887), and *Thoradonta nodulosa* (Stål, 1861). DNA barcoding revealed that color morphs are genetically similar, and morphometric analysis likewise showed no distinct clustering. Together, these results indicate that different color morphs indeed represent intraspecific polymorphisms. More importantly, we demonstrate that combining molecular data with morphological evidence offers an effective, integrative framework for resolving taxonomic challenges within tetrigids and highlights the value of integrative approaches for clarifying species boundaries in morphologically variable taxa.

## ﻿Introduction

Color polymorphism is the existence of two or more distinctly colored phenotypes within an interbreeding population (Huxley 1955). This phenomenon is ubiquitous in animals (McKinnon and Pierotti 2010; Wellenreuther et al. 2014) and can be subject to both natural and sexual selection, including predator–prey interactions and mate preferences (e.g., Roulin and Wink 2004; Roulin and Bize 2007; Sánchez-Guillén et al. 2013). Investigation of its genetic basis and phenotypic interactions with the environment has offered us insights into evolution, including the mechanisms that maintain such polymorphism and its role in speciation (Hugall and Stuart-Fox 2012). In systematics, color polymorphism can correlate with phylogenetic relatedness and/or relate to geographical and environmental variations (Drosopoulos 2003; Messmer et al. 2005; Beukema et al. 2016). Consequently, color variations among insect taxa have always intrigued systematists and taxonomists because they are keen to ascertain whether such variations represent intraspecific polymorphism or distinct species differences (Honek and Furlan 1995; Nie et al. 2012; Blow et al. 2021; Ramaiah et al. 2023). Polymorphism can also complicate or lead to erroneous species delimitation. This has led to integrative approaches incorporating life history, morphology and phylogeny being adopted to address this question, in various insect groups including coleopterans, hemipterans, and odonates (e.g., Nie et al. 2012; Blow et al. 2021; Ramaiah et al. 2023), as well as orthopterans (Cueva Del Castillo et al. 2021; Fianco 2023).

Understanding of color variability remains limited, however, in numerous insect taxa. Among these are the pygmy grasshoppers (Orthoptera: Tetrigidae), a monophyletic clade representing the most primitive family within the suborder Caelifera (Song et al. 2015). Color polymorphism is extensive among tetrigids (Nabours 1929; Nabours and Foster 1929; Holst 1986; Ichikawa et al. 2006; Zhao et al. 2016), including species from Southeast Asia (Forsman 2000; Adžić 2021; Neo et al. 2023). Among tetrigid species known to display color polymorphism, such as *Tetrix japonica* (Kaori et al. 2010), *Tetrix undulata* (Ahnesjö and Forsman 2003), and *Tetrix subulata* (Tetriginae: Tetrigini) (Forsman and Appelqvist 1998; Forsman 1997, 2000), polymorphism in color and patterns can be discrete and/or continuous.

Despite the widespread reports of color polymorphism in tetrigids, few studies have rigorously tested species delimitation of these color polymorphic taxa using an integrative taxonomic approach. Integrative taxonomy refers to the practice of incorporating multiple lines of evidence, including morphology and genetics, to delineate species in taxonomy. There is a pressing need to continue apply integrative taxonomic approaches to tetrigids, as they have been shown to effectively distinguish intraspecific color and pattern variations from species-specific differences in the family (Zhao et al. 2016). Historically, color morphs have often been mistaken to be distinct species (e.g., Bolívar 1887; Hancock 1907; Hancock 1909; Günther 1939; Devriese 1996; Long et al. 2023). This misidentification largely arose because, unlike other orthopterans, tetrigids lack reliable diagnostic morphological characters (Tumbrinck 2014; Tumbrinck and Skejo 2017; Muhammad et al. 2018; Zha et al. 2021; Skejo et al. 2022). Specifically, the male genitalia, which are often sclerotized and bear many reliable taxonomic characters used for diagnosing orthopteran species (Song 2010; Chamorro-Rengifo and Lopes-Andrade 2014; Gorochov 2015), typically do not offer diagnostic features for tetrigids. This calls for the use of a more integrative approach, considering additional lines of evidence, such as molecular or morphometric data.

In integrative taxonomy, morphometric and genetic data are frequently used as complementary lines of evidence to attain a more holistic understanding of species delimitations in the taxa of interest. Morphometric analysis provides a more quantitative approach for examining morphology and has recently emerged as a rigorous approach for delimiting species within tetrigids (Tumbrinck 2014; Tan et al. 2017; Pan et al. 2018; Tan et al. 2019; Moser et al. 2021). This method is particularly useful for cryptic species exhibiting subtle morphological differences that are difficult to discern visually without quantitatively measuring and comparing characters (Tan et al. 2017). Traditional morphometric approaches involve measuring body part lengths and comparing their ratios (Pan et al. 2018; Moser et al. 2021), whereas more recent methods employ distance-based cluster analyses such as Principal Component Analysis (PCA) and neighbor-joining (NJ) trees. These analyses identify possible diagnostic characters influenced by speciation, and work on the assumption that all body measurements are interrelated (Tan et al. 2017; Pan et al. 2018; Tan et al. 2019; Moser et al. 2021).

The application of genetic data to objectively test species delimitation hypotheses in tetrigids has emerged recently (e.g., Zhao et al. 2016; Tan et al. 2017; Kasalo et al. 2025; Fite et al. 2025). DNA barcodes, such as the *cytochrome c oxidase subunit I* (*COI*), have been used to construct gene trees to infer species delimitations (Kasalo et al. 2023, 2025; Fite et al. 2025). These above-mentioned studies, however, have largely excluded color-variable taxa. Integrating DNA barcode information could greatly enhance tetrigid species delimitation, especially since coalescent-based methods have statistical rigor and offer an additional approach to determine whether color morphs observed represent within-species color polymorphisms or distinct species.

**Figure 1.**
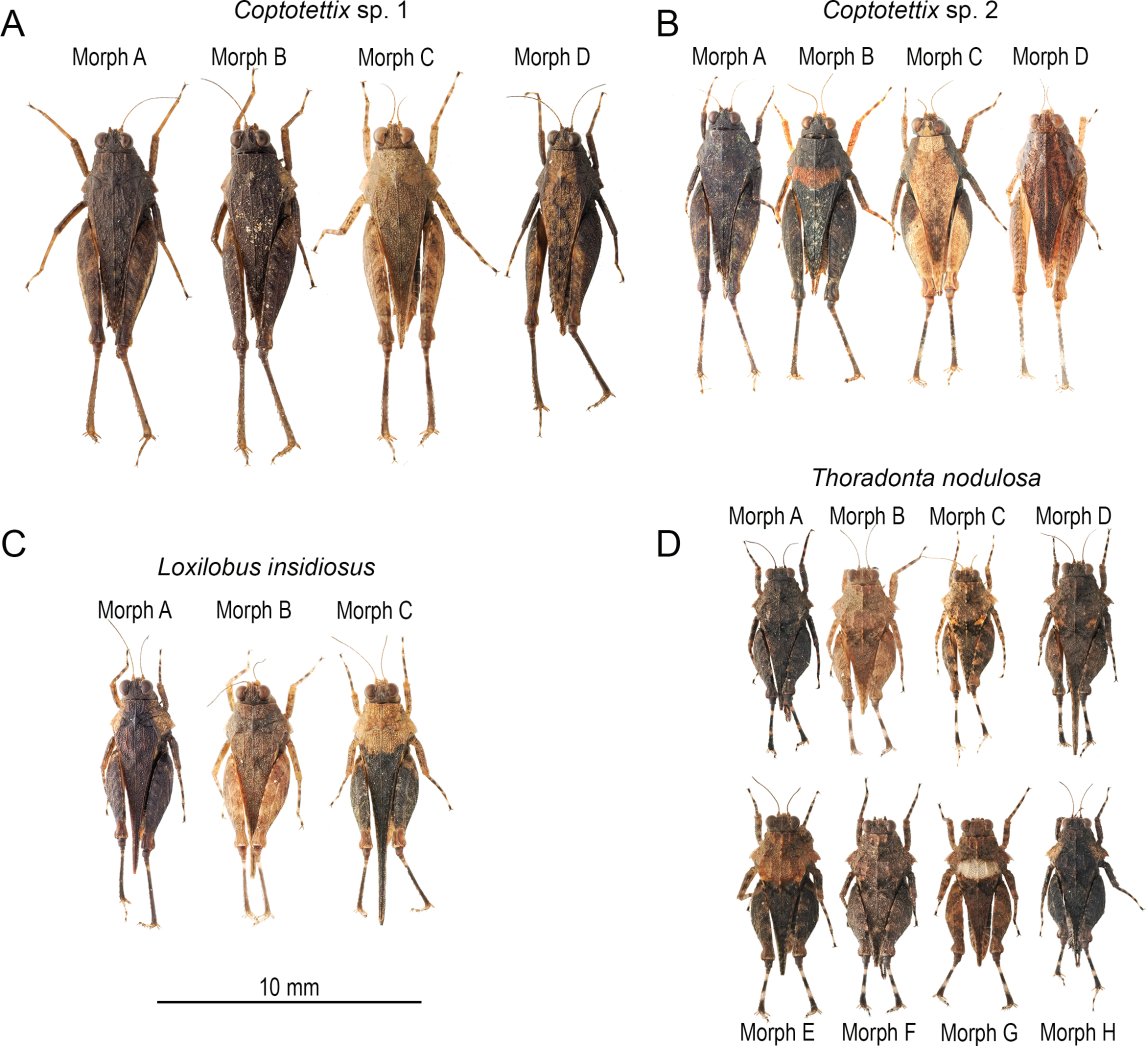
Dorsal view of the different color morphs. A. *Coptotettix* morphospecies 1; B. *Coptotettix* morphospecies 2; C. *Loxilobus insidiosus*; D. *Thoradonta nodulosa*.

In recent surveys, we observed color variations in four putative species identified as *Coptotettix* morphospecies 1, *Coptotettix* morphospecies 2, *Loxilobus insidiosus* (Bolívar, 1887) (Criotettiginae: Thoradontini), and *Thoradonta nodulosa* (Stål, 1861) (Criotettiginae: Thoradontini) (see Neo et al. 2023) (Fig. 1) collected from the Nee Soon freshwater swamp forest in Singapore (Neo et al. 2023). Neo et al. (2023) postulated that these color morphs are probably intraspecific variants based on current taxonomic literature, but did not test this hypothesis via an integrative taxonomic approach. By employing both DNA barcodes and morphometry, we test whether the color variations in these taxa are indeed distinct species or represent interspecific color polymorphism. Putative species units were obtained using DNA barcodes (i.e., molecular operational taxonomic units or mOTUs) using two mitochondrial gene fragments: *COI* and *small ribosomal RNA subunit III* [*12S*]). Molecular species delimitation was done using an objective clustering method (following the principles of SpeciesIdentifier, see Meier et al. 2006), as well as a coalescent-based species delimitation method (i.e., Assemble Species by Automatic Partitioning analysis, see Puillandre et al. 2021). We also performed a morphometric distance-based cluster analysis using 22 characters. We are hitherto not aware of any study that uses such an integrative approach to address the color variability in tetrigids in Southeast Asia. By using color polymorphic taxa as a case study, we aim to show how integrative taxonomy can quickly help resolve taxonomic problems in tetrigids, including over-splitting of polymorphic taxa.

## ﻿Materials and methods

### ﻿Material examined and identification

Between November 2022 and February 2023, tetrigids were opportunistically collected from Nee Soon Swamp Forest (NSSF), Singapore (Suppl. material 1). Permission to conduct faunistic surveys and collection of material was granted by the National Parks Board (NP/RP22-096 and NP/RP22-113). Specimens were imaged for morphometric measurements and identification (see Neo et al. 2023), then stored in −80 °C freezer. The grasshoppers were identified as putative species, using photographs and descriptions of type specimens, comparison with other specimens in the Zoological Reference Collection (**ZRC**) of the Lee Kong Chian Natural History Museum (**LKCNHM**), as per Neo et al. (2023). When color variations were observed in the putative species, we sorted them into different color morphs:

For *Coptotettix* morphospecies 1 (Fig. 1A):

A: Uniformly dark pronotal coloration, pale femur stripe (when viewed laterally)

B: Dark pronotal coloration, pale lateral lobes

C: Uniformly pale pronotal coloration, banded femur (when viewed laterally)

D: Pale pronotal coloration, dark lateral lobes

For *Coptotettix* morphospecies 2 (Fig. 1B):

A: Uniformly dark pronotal coloration

B: Dark pronotal coloration, pale dorsal band across center of pronotum

C: Pale pronotal coloration, dark lateral lobes

D: Pale pronotal coloration, multiple dorsal stripes along center of pronotum

For *Loxilobus insidiosus* (Fig. 1C):

A: Dark pronotal coloration, pale lateral lobes

B: Uniformly pale pronotal coloration

C: Half pale (anterior) half dark (posterior) pronotal coloration

For *Thoradonta nodulosa* (Fig. 1D):

A: Uniformly dark pronotal coloration

B: Uniformly pale pronotal coloration

C: 3 pale pronotal patches (1 posterior to head, and 2 mid lateral of pronotum)

D: 2 pale pronotal patches (2 mid lateral of pronotum)

E: Half pale (anterior) half dark (posterior) pronotal coloration

F: 1 pale pronotal patch posterior to head

G: Pale pronotal coloration, paler band across pronotum

H: Dark pronotal coloration, pale lateral lobes

In our dataset, we also included two non-polymorphic but morphologically cryptic species, i.e., *Loxilobus neesoon* and *Loxilobus simulans* (Criotettiginae: Thoradontini). These two species will serve as a control and test-bed for both the species delimitation and morphometric analysis. We expect that both analyses will reveal these as two distinct taxa.

### ﻿Morphometric measurements

Measurements of 22 morphological characters were made for each specimen, whenever possible, as described in Neo et al. (2023) (Suppl. material 1). Each specimen was photographed and digitally measured using ImageJ 1.53t (Wayne Rasband, Research Services Branch, National Institute of Mental Health, Bethesda, MD, USA) (Neo et al. 2023: table 1). For the measurements, the abbreviations of the characters follow those in Neo et al. (2023):

**PL** Pronotal disc length

**PW** Pronotum disc width

**PLW** Pronotal lateral lobe width

**VW** Vertex width

**EW** Eye width

**PAM** Width of anterior margin of pronotal disc

**PCW** Prozonal carina width

**PCL** Prozonal carina length

**PH** Pronotum height

**IAH** Infra-scapular area height

**TW** Tegmen width

**PTL** Posterior tibia length

**PFL** Posterior femur length

**PFW** Posterior femur width

**FHW** Fastigial horn width

**SW** Scutellum width

**CEH** Clypeus-eye height

**AGH** Antennal groove-eye height

**PML** Pronotum anterior margin-notch length

**NW** Notch width

**ICL** Interhumeral carina length

**CW** Carina width

### ﻿Morphometric analysis

To examine how color morphs may differ morphologically, measurements taken for putative species *Coptotettix* morphospecies 1, *Coptotettix* morphospecies 2, *Loxilobus insidiosus*, and *Thoradonta nodulosa* were summarized into major gradients of variation by performing PCA on scaled measurements using the ‘pca.calc’ function in the R package ‘MorphoTools2’ (Šlenker et al. 2022). Biplots were then observed for any clustering or driving traits differentiating the color morphs using the functions ‘plotPoints’ and ‘plotAddSpiders’. We also tested whether PCA of the morphometric measurements can differentiate well-established cryptic species pair *Loxilobus simulans* and *Loxilobus neesoon*. Previously, Tan et al. (2017) also employed morphometry to separate these two cryptic species.

### ﻿Molecular protocols: DNA barcoding, sequencing, and bioinformatics

DNA extraction was done using the QuickExtract DNA Extraction Solution (ScientificResources), which allows for rapid DNA extraction by incubation alone. DNA template was obtained from specimens by plucking a leg, submerging it in QuickExtract solution, and incubating at 65 °C for 18 min, followed by 98 °C for 2 min.

PCR amplification was conducted using two sets of primers: universal metazoan *COI* primers mICOIintF: 5’- GGWACWGGWTGAACWGTWTAYCCYCC -3’ (Leray et al. 2013) and jgHCO2198: 5’- TAIACYTCIGGRTGICCRAARAAYCA -3’ (Geller et al. 2013) which targeted and amplified the 313 bp *COI* mini-barcode, the *12S*F: 5’- TACTATGTTACGACTTAT -3’ and *12S*R: 5’- AAACTAGGATTAGATACCC -3’ (Chintauan-Marquier et al. 2016; Kambhampati, 1995). These primers were tagged with 13 bp indices, and possessed at least 4 base-pair differences and lacked homopolymers (Srivathsan et al. 2021), making them suitable for MinION sequencing. Each PCR sample was assigned a unique combination of forward and reverse primer tags, which will facilitate downstream bioinformatic assignment to specimens. Negative controls, where DNA extract was replaced with molecular-grade water, were included for each batch of (at most) 95 PCR samples.

Regardless of the primer used, each PCR sample volume contained 8 µL of CWBio 2× Master Mix, 1 µL of 1 mg/mL BSA, 2 µL of 5 µM of each primer, and 3 µL of DNA extract (10× diluted). The thermocycling conditions varied for each gene fragment: for *COI*, a starting denaturation step of 5 min at 94 °C, followed by 40 cycles of 30 s at 94 °C, 45 s at 45 °C and 45 s at 72 °C, and a final extension step of 5 min at 72 °C; for *12S*, a step-up reaction starting with a denaturation step of 5 min at 94 °C, followed by 10 cycles of 30 s at 94 °C, 30s at 45 °C and 45 s at 72 °C, then 40 cycles of 30 s at 94 °C, 30 s at 47 °C and 45 s at 72 °C, and a final extension step of 5 min at 72 °C. After amplification, PCR products for the same gene fragment were pooled in equal volumes and purified using Ampure XP beads (Beckman Coulter, USA) as per manufacturer’s instructions. The pools were then quantified using the dsBR kit on the Qubit fluorometer and equimolar combined.

Library preparation was conducted using the Ligation Sequencing Kit V12 (SQK-LSK112), and sequencing was done on the MinION mk1b. Although Kit V12 was used, we followed the library preparation protocol “Ligation Sequencing Amplicons V14”, with one modification: during End-prep, DNA CS was replaced with molecular-grade water.

Raw FAST5 data was base-called using Guppy and the r10.3 super accurate model. The resulting FASTQ files were demultiplexed using ONTbarcoder (v. 0.1.9) (Srivathsan et al. 2021). Different quality check (QC) filters were applied to each gene fragment. For *COI*, the inbuilt filters of ONTbarcoder were applied, targeting protein-coding genes. Sequences with ambiguous bases were discarded, as we found that they would later interfere with pairwise-distance (p-distance) calculations for mOTU (molecular operative taxonomic units) clustering. The remaining sequences were uploaded to the GBIF’s online search engine (https://www.gbif.org/tools/sequence-id) to get a preliminary taxonomic assignment. Only sequences matching ≥ 85% to tetrigids were retained, while others were treated as contaminated samples and discarded.

For *12S*, which is not a protein-coding gene, the inbuilt QC filters of ONTbarcoder could not be applied. Post-demultiplexing, sequences with ambiguous bases were discarded. The remaining sequences were queried against the GenBank and DNA barcode database using MegaBLAST from BLAST 2.13.0+ (Camacho et al. 2009) to attain a preliminary taxonomic assignment. Only sequences matching ≥ 85% to tetrigids were retained, while the rest were discarded.

For selected specimens, where DNA barcodes were not obtained, PCR amplification was repeated, and PCR products were sent to Macrogen Asia Pacific Private Limited for Sanger sequencing. The same PCR reagents and protocols described above were used, except that the PCR mastermix was replaced with the Vazyme 2× Rapid Taq Master Mix P222. Chromatographs of DNA sequences were viewed and trimmed using the default settings in Geneious Prime 2023.2.1 (Java Version 11.0.18). Sanger sequencing results revealed that for the following taxa and genes, the earlier blanket thresholds we used for flagging contaminations (i.e., ≥ 85% match to tetrigids) was too stringent: *Coptotettix* morphospecies 1A, *Thoradonta nodulosa* morph E and *Potua coronata* for *COI*; *Loxilobus simulans* and *Potua coronata* for *12S*. These species and DNA barcodes sequences were absent from the existing online databases and did not match ≥ 85% to the existing tetrigid sequences available, even though it turned out that the DNA barcode sequence attained from both sequencing methods (MinION and Sanger sequencing) were exactly the same. For these taxa and genes, we made an exception and readed them into our dataset.

The sequences generated for this study were deposited in GenBank with accession numbers PP760259–PP760335 for *COI* and PP778511–PP778662 for *12S* (Suppl. materials 2, 3, respectively).

### ﻿Species delimitation

To cluster specimens into putative species according to their DNA barcodes, we used an objective clustering program, which relies on pairwise-distances (p-distance), as well as the principles of SpeciesIdentifier (Meier et al. 2006). Objective clustering was performed for each gene (i.e., *COI* and *12S*) separately. A dendrogram was built for each gene, starting with the smallest pairwise distances. Subsequently, a coalescent-based species delimitation analysis known as the ASAP (Puillandre et al. 2021) was employed for each of the single locus sequence alignments, to determine the putative species units among tetrigid specimens. ASAP divides species partitions based on pairwise genetic distances, and computes a probability of panmixia (p-val), a relative gap width metric (W) and ranked results by the asap-score: the lower the score, the better the partitioning (Puillandre et al. 2021). Sequence alignments were done using MAFFT v. 7, through the web-server accessible at https://mafft.cbrc.jp/alignment/server/. The ASAP analysis was executed using the Kimura K80 substitution model (ts/tv = 2.0) through the web-server accessible at https://bioinfo.mnhn.fr/abi/public/asap.

### ﻿Phylogenetic analysis

To conduct a preliminary examination of relationships between species, which cannot be accomplished using species delimitation analysis, we ran a phylogenetic analysis for *COI* with maximum likelihood (ML) using the using the iq-tree web server with the default options (http://iqtree.cibiv.univie.ac.at/; Trifinopoulos et al. 2016). We emphasize that this analysis is preliminary, as it relies primarily on short COI fragments, and should only be interpreted as a hypothesis to guide future, more comprehensive studies. We also combined our data with closely related or similar species (i.e., congenerics) from the region in which *COI* sequences are available on public depositories such as GenBank and BOLD. In total, 102 sequences were included in this preliminary phylogenetic analysis. We used ModelFinder to search for the best model among 88 available DNA substitution models. The best model was chosen according to Bayesian Information Criterion (BIC). Branch support was assessed by conducting 100 standard bootstrap replicates. Branches with bootstrap support values (BS) ≥ 75% were considered strongly supported.

## ﻿Results

### ﻿Species delimitation

**Figure 2.**
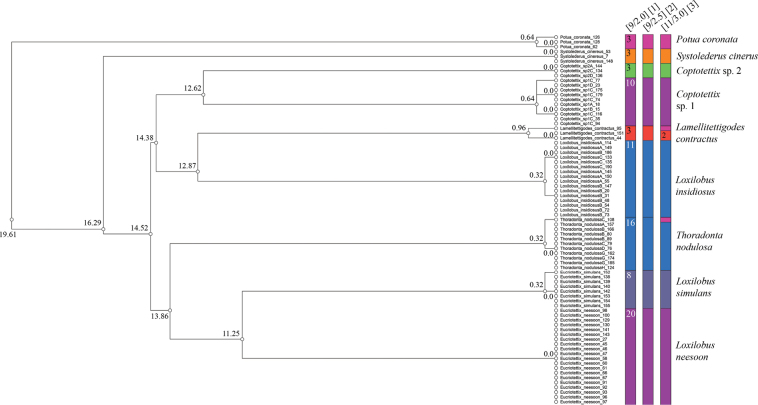
Dendrogram from objective clustering and the first–third best species partitions from ASAP analyses of *COI* sequences of 76 specimens belonging to nine putative species. The values on the branches of the dendrogram represent the genetic distance. For ASAP, the header [x, y] represents the number of species partitions and the asap score; and the number in each species partition represents the number of specimens grouped within the delimited species.

Based on *COI* sequences of 76 specimens belonging to nine putative species, both the objective clustering analysis and ASAP revealed that *Coptotettix* morphospecies 1, *Coptotettix* morphospecies 2, *Loxilobus insidiosus*, and *Thoradonta nodulosa* can each be considered as a single putative species (Fig. 2, Suppl. material 4). For instance, in most insects, pairwise differences larger than 3% in the *COI* barcode are often used as a threshold to delineate separate species (Meier et al. 2016; Wang et al. 2018; Yeo et al. 2020). The lowest genetic distances between the nine putative species range from 11.25–19.61%, demonstrating that there is a clear gap in interspecific genetic distances. *Coptotettix* morphospecies 1, *Coptotettix* morphospecies 2 also differed by a p-distance of at least 12.62%. In contrast, the most genetically divergent specimen within morphospecies 1 had p-distance of as low as 0.64% to one other specimen in the cluster, and for *Loxilobus insidiosus* and *Thoradonta nodulosa* the p-distance is 0.32% and 0.32%, respectively. After a global-alignment, the applied ASAP procedure identified nine mOTUs (hypothetical species) at the threshold distance 7.35% which has the best asap-score (2.0), and a second partition with identical results and inference. DNA data for *Coptotettix* morphospecies 2 color morph C was available and was genetically similar to other color morphs, although no *COI* data was available for morph B.

**Figure 3.**
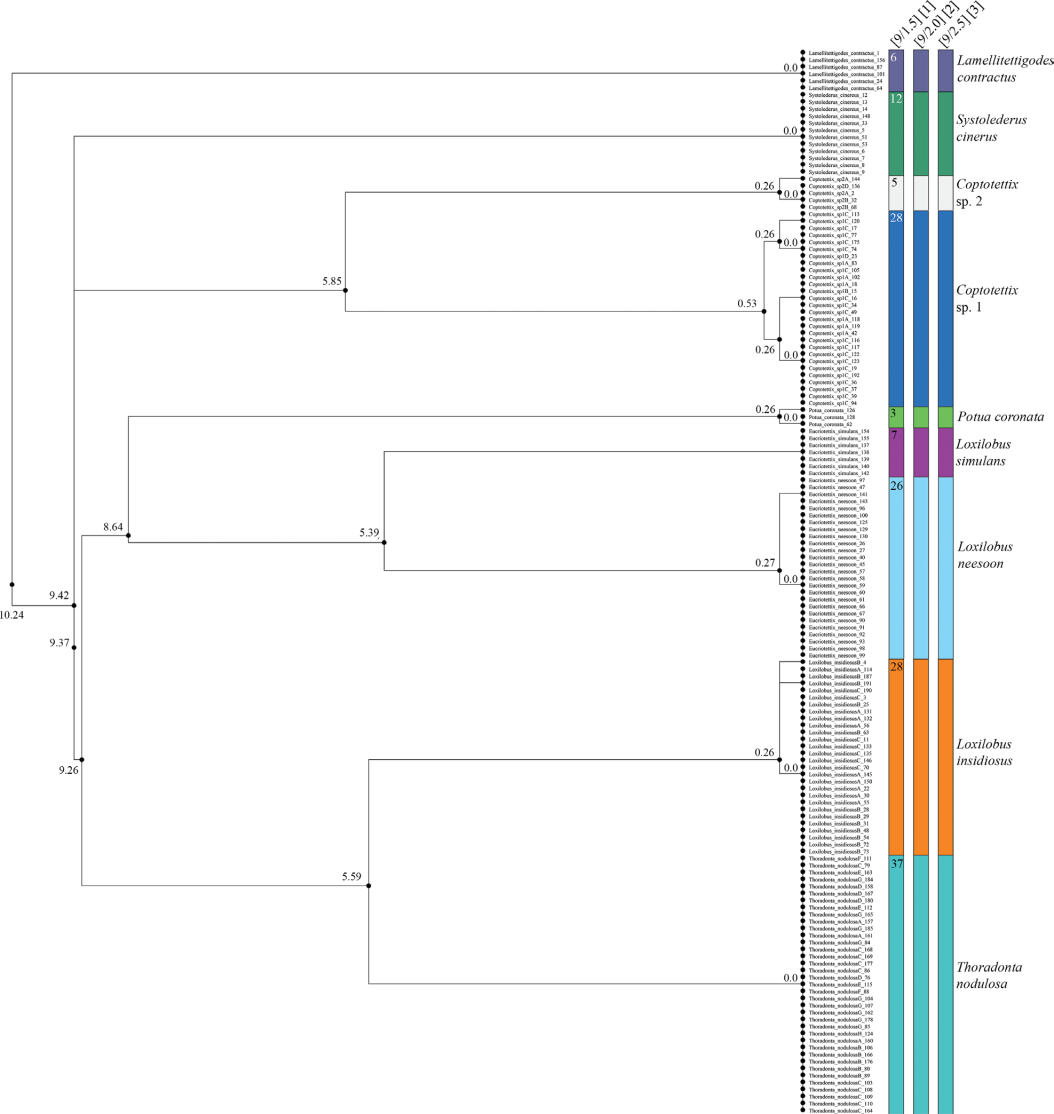
Dendrogram from objective clustering and the first–third best species partitions from ASAP analyses of *12S* sequences of 152 specimens belonging to nine putative species. The values on the branches of the dendrogram represent the genetic distance. For ASAP, the header [x, y] represents the number of species delimited and the asap score; and the number in each partition represents the number of specimens grouped within the delimited species.

Likewise, based on *12S* sequences of 152 specimens belonging to nine putative species, both the objective clustering analysis and ASAP revealed that color morphs observed in *Coptotettix* morphospecies 1, *Coptotettix* morphospecies 2, *Loxilobus insidiosus* and *Thoradonta nodulosa* are genetically similar, and can each be considered as a single species (Fig. 3, Suppl. material 5). Between the nine putative species, the lowest genetic distances range from 5.38–10.24%, once again demonstrating a clear gap in interspecific genetic distance. *Coptotettix* morphospecies 1 and *Coptotettix* morphospecies 2 were genetically very different: between them, specimens had p-distance of at least 5.85%. On the other hand, specimens within morphospecies 1 were split into two genetic clusters, but there was at least one pair of specimens with as low as 0.53% p-distance between these clusters. Similarly, for each of *Coptotettix* morphospecies 2 and *Loxilobus insidiosus*, the last specimen with the highest genetic distance from all other specimens had at least 0.26% p-distance from one other specimen in their respective clusters. For *Thoradonta nodulosa*, all the specimens in our dataset had the same haplotype. After a global-alignment, the applied ASAP procedure identified nine mOTUs (hypothetical species) at the threshold distance 4.10% which has the best asap-score (1.5). The ASAP results showed that the first- (lowest asap score), second- (next lowest asap score) and third-best partitions show identical results, and corroborates the mOTUs delimitation from objective clustering analysis.

### ﻿Morphometric analysis

**Figure 4.**
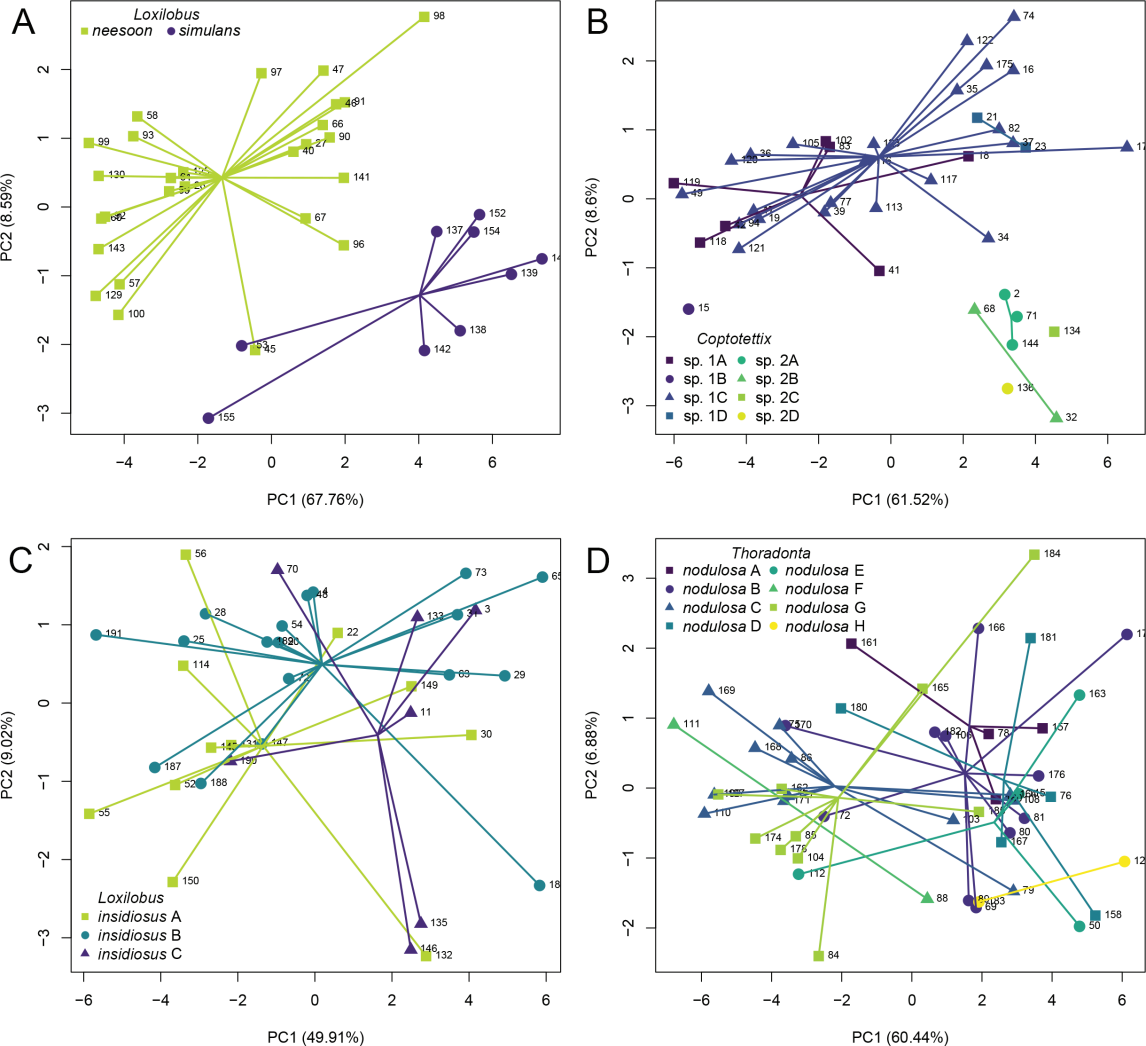
Plots of Principal Component Analysis for morphometry of. A. *Loxilobus simulans* and *Loxilobus neesoon*; B. *Coptotettix* spp.; C. *Loxilobus insidiosus*; D. *Thoradonta nodulosa*.

As a proof-of-concept using the two *Loxilobus* cryptic species which do not exhibit color polymorphism, we performed a PCA based on 20 characters (ICL and ICW are not available for the genus) from 27 *Loxilobus neesoon* (Tan & Storozhenko, 2018) and nine *Loxilobus simulans* (Tan & Storozhenko, 2017) specimens. The biplot yields the first two components explaining 76.4% of variance, and the two species show two distinct clusters (Fig. 4A).

Following this, we performed a similar PCA for *Coptotettix* morphospecies 1 and *Coptotettix* morphospecies 2 based on 20 characters from 33 morphospecies 1 and seven morphospecies 2 specimens, which accounts for 70.1% of variance. ICL and ICW were not available for all specimens. The two morphospecies form two distinct clusters, but the color morphs within each morphospecies do not (Fig. 4B). This is most prominent among A, C and D color morphs of morphospecies 1. There was only one specimen for B color morph of morphospecies 1 which did not appear to cluster with the other color morphs. For morphospecies 2, owing to the limited number of specimens, it is not so obvious that the four color morphs overlap in their morphometry. However, it is notable that variations among the color morphs within each morphospecies are smaller than between both morphospecies.

For *Loxilobus insidiosus*, the PCA based on all 22 characters from 36 specimens, which accounts for 58.9% of variance, shows that the three color morphs were indistinguishable (Fig. 4C). Likewise, for *Thoradonta nodulosa*, the PCA based on all 22 characters from 50 specimens, which accounts for 67.3% of variance shows that the eight color morphs were morphometrically similar (Fig. 4D).

### ﻿Phylogenetic analysis

**Figure 5.**
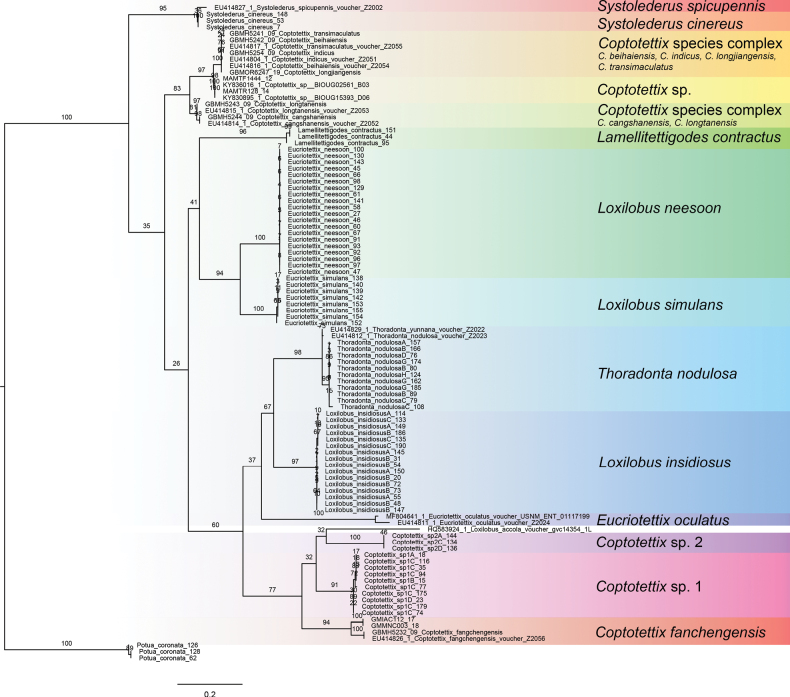
Maximum Likelihood (ML) tree showing the phylogeny of *COI* of Tetrigidae. Support values for ML analyses are given at branches (BS); BS values below 75% are considered as low support.

ML analysis for the *COI* sequences was done using the best-fit model according to BIC, i.e., Transitional Model of Molecular Evolution (TIM2)+F+I+Gamma distribution (G4) (Fig. 5). Most species were recovered as monophyletic groups (all with at least BS of 90%). The only exceptions are the *Coptotettix* comprising of *Coptotettix beihaiensis* Deng & Zheng, 2006, *Coptotettix indicus* Hancock, 1912, *Coptotettix longjiangensis* Zheng & Wei, 2000, and *Coptotettix transimaculatus* Zheng & Jiang, 2002, as well as *Coptotettix cangshanensis* Zheng, Nie & He, 2005 and *Coptotettix longtanensis* Zheng & Jiang, 2004. Generally, closely related species were recovered as distinct monophyletic groups. These include the two morphologically cryptic *Loxilobus* (i.e., *Loxilobus neesoon* and *Loxilobus simulans*) (BS of 94%) and two species of *Systolederus* (BS of 95%). Deeper relationships were generally poorly supported (BS of ≤ 75%), such as potential sister lineages made up of *Loxilobus insidiosus* and *Thoradonta*.

## ﻿Discussion

Our study on the Tetrigidae from Singapore demonstrates the use of integrative taxonomy to test species-delimitation hypothesis of polymorphic species by incorporating genetic and morphometric data, and can be applied to other tetrigids worldwide. We have also sequenced the first DNA barcode for a small and rare genus, *Potua* (Cladonotinae, Xerophyllini) from Singapore. Species delimitation using *COI* and *12S* loci as well as morphometric analyses of 20 to 22 morphometric characters, unequivocally showed that well-established cryptic species pair *Loxilobus simulans* and *Loxilobus neesoon* can be effectively differentiated. It also demonstrates that color variations observed in *Coptotettix* morphospecies, *Thoradonta nodulosa*, and *Loxilobus insidiosus* do not indicate species differences. Specifically, the color morphs of *Coptotettix* morphospecies 1 (four morphs), *Coptotettix* morphospecies 2 (four morphs), *Thoradonta nodulosa* (three morphs), and *Loxilobus insidiosus* (eight morphs) have very low intraspecific genetic variability, and are inseparable by morphometry. Intraspecific color variability is widely documented among tetrigids (Holst 1986; Ichikawa et al. 2006; Zhao et al. 2016; Adžić 2021; Long et al. 2023). Within Singapore, for example, color variability has been observed in other tetrigids species, such as *Paratettix variabilis* Bolívar, 1887, *Paratettix curtipennis* (Hancock, 1912) (Tetriginae: Tetrigini), *Ergatettix dorsiferus* (Walker, 1871) (Tetriginae), and *Criotettix robustus* (Hancock, 1907) (Criotettiginae: Criotettigini) (see Tan, 2012). Therefore, in a broader context, our findings can probably apply to other tetrigids exhibiting color morphs, suggesting that any color variations should not be interpreted as species differences first unless otherwise suggested by molecular and/or morphometry evidence.

Intraspecific color polymorphism observed in these pygmy grasshoppers is likely driven by selection pressure, with certain color morphs exhibiting better camouflaging abilities in specific microhabitats (Neo et al. 2023). For instance, Neo et al. (2023) observed that light-colored morphs of *Thoradonta nodulosa* tend to be found among leaf litter and herbaceous cover, whereas dark-colored morphs are prevalent in exposed microhabitats. This also aligns with the findings of Zha et al. (2016), which showed that body colorations in *Thoradonta* are adapted to their local substrate. However, the genetic basis of the color polymorphism occurring in *Coptotettix* species, *Thoradonta nodulosa*, and *Loxilobus insidiosus* remains unanswered. Further in-depth genomic investigations may be necessary to determine if there is a genetic basis to this color polymorphism.

Incorporating DNA data into future taxonomic work for tetrigids will be important to corroborate species hypothesis from non-reproductive characters, which have been used predominantly to delimit species. At present, the utilization of DNA data for taxonomic studies of tetrigids is very limited. A quick survey of the DNA barcode data available online (data sourced 22 November 2022) found that a great proportion of the data deposited is not identified properly to species or genus levels (Kasalo et al. 2023). The study by Zhao et al. (2016) on *Tetrix bolivari* represents one of the rare instances in which DNA barcoding was used to examine explicitly color polymorphism in pygmy grasshoppers. However, Lehmann et al. (2017) later pointed out that Zhao et al. (2016) had misidentified their study subject, and it should have been a *Paratettix* species. This further reiterates that good alpha taxonomy remains crucial in building reliable DNA barcode databases, which is currently still lacking for tetrigids. Consequently, we also followed the advice of Lehmann et al. (2017), by ensuring that images showing the morphology of the taxa of interest are available (see Neo et al. 2023). We also ensured that the specimens with DNA barcodes are vouchered properly and deposited in an accessible repository for future alpha taxonomic validation and revision, whenever necessary.

The molecular species delimitation using *COI* and *12S* loci can be a reliable tool to identify species, however, it should not be used to infer evolutionary relationships. To provide a very preliminary hypothesis of species relationships, we constructed a phylogenetic tree using predominantly short COI fragments, which generally supported our species delimitation (Fig. 5). We would like to emphasize again that the dendrograms in Figs 2, 3 were generated for both the *COI* and *12S* loci from objective clustering analysis and ASAP and are based on pairwise distances, instead of phylogenetic relationships based on genetic models. Hence, caution should be taken not to use it to make inferences on evolutionary relationships. Preliminary phylogenetic analysis of *COI* sequences (Fig. 5) recovered most species as well-supported monophyletic groups, with the exception of some *Coptotettix* species. Closely related species, including morphologically cryptic taxa, were generally recovered as distinct monophyletic groups. However, the ML tree offers limited insight into deeper evolutionary relationships, as the evolution of one locus does not necessarily represent species evolution and has reduced resolution for deeper nodes (Hajibabaei et al. 2007). Additional biases may arise from the mitochondrial origin of COI owing to incomplete lineage sorting and maternal inheritance (Moritz and Cicero 2004). We recommend that future studies use multiple loci from both mitochondrial and nuclear genomes to make robust phylogenetic inferences (Moritz and Cicero 2004; Hajibabaei et al. 2007; Li et al. 2021, 2025a, 2025b) and to treat our results as preliminary hypotheses.

Morphometric analyses have been shown to offer a more quantitative approach to delimit species of tetrigids, including discovering cryptic species (Tan et al. 2017). However, care should be taken to properly differentiate between intraspecific and interspecific differences. For example, some species, including *Loxilobus insidiosus* and *Thoradonta nodulosa* exhibit huge intraspecific variation in their pronotum sizes and hind wing lengths. Individuals with different pronotum and hind wing lengths can and have misled taxonomists to erroneously consider them as distinct species (Long et al. 2023). We also observed that deformities in the pronotum are not uncommon, and this can sometimes be natural or an artefact of specimen preservation (Long et al. 2023). It is therefore crucial that these abnormalities should not be used for the analysis to avoid biases or misinterpretation.

## ﻿Conclusions

Our study highlights the utility and importance of integrative taxonomy for tetrigids. Using alpha taxonomy, we identified color variants observed in four putative species—*Coptotettix* morphospecies 1, *Coptotettix* morphospecies 2, *Loxilobus insidiosus*, and *Thoradonta nodulosa*—collected from a single site in Singapore. Using objective clustering and species delimitation of DNA barcodes, as well as morphometric analysis, we established that discontinuous color variations observed are merely intraspecific variations (color polymorphism) rather than representing different species. This approach can also be used to resolve taxonomic problems in tetrigids.

## ﻿Additional information

### Use of AI

No use of AI was reported.

### Author ORCIDs

Ivan Neo https://orcid.org/0009-0006-8729-3847

Rebecca Ker Loh https://orcid.org/0000-0001-9522-6843

Tricia J. Y. Cho https://orcid.org/0009-0004-5332-8154

Nalini Puniamoorthy https://orcid.org/0000-0003-0651-8356

Darren C. J. Yeo https://orcid.org/0000-0003-2669-4349

Ming Kai Tan https://orcid.org/0000-0002-4324-6305

### Data availability

All of the data that support the findings of this study are available in the main text or Supplementary Information.

## Supplementary materials

Supplementary material 1 Material examined Data type xlsx https://binary.pensoft.net/file/1454456This dataset is made available under the Open Database License (http://opendatacommons.org/licenses/odbl/1.0/). The Open Database License (ODbL) is a license agreement intended to allow users to freely share, modify, and use this Dataset while maintaining this same freedom for others, provided that the original source and author(s) are credited.Ivan Neo, Rebecca Ker Loh, Tricia J. Y. Cho, Nalini Puniamoorthy, Darren C. J. Yeo, Ming Kai Tan

Supplementary material 2 COI sequences Data type txt https://binary.pensoft.net/file/1454457This dataset is made available under the Open Database License (http://opendatacommons.org/licenses/odbl/1.0/). The Open Database License (ODbL) is a license agreement intended to allow users to freely share, modify, and use this Dataset while maintaining this same freedom for others, provided that the original source and author(s) are credited.Ivan Neo, Rebecca Ker Loh, Tricia J. Y. Cho, Nalini Puniamoorthy, Darren C. J. Yeo, Ming Kai Tan

Supplementary material 3 12S sequences Data type txt https://binary.pensoft.net/file/1454458This dataset is made available under the Open Database License (http://opendatacommons.org/licenses/odbl/1.0/). The Open Database License (ODbL) is a license agreement intended to allow users to freely share, modify, and use this Dataset while maintaining this same freedom for others, provided that the original source and author(s) are credited.Ivan Neo, Rebecca Ker Loh, Tricia J. Y. Cho, Nalini Puniamoorthy, Darren C. J. Yeo, Ming Kai Tan

Supplementary material 4 Pair-wise distance of COI sequences Data type csv Explanation note Table showing pair-wise distance of COI sequences between specimens studied here. https://binary.pensoft.net/file/1454459This dataset is made available under the Open Database License (http://opendatacommons.org/licenses/odbl/1.0/). The Open Database License (ODbL) is a license agreement intended to allow users to freely share, modify, and use this Dataset while maintaining this same freedom for others, provided that the original source and author(s) are credited.Ivan Neo, Rebecca Ker Loh, Tricia J. Y. Cho, Nalini Puniamoorthy, Darren C. J. Yeo, Ming Kai Tan

Supplementary material 5 Pair-wise distance of 12S sequences Data type csv Explanation note Table showing pair-wise distance of 12S sequences between specimens studied here. https://binary.pensoft.net/file/1454460This dataset is made available under the Open Database License (http://opendatacommons.org/licenses/odbl/1.0/). The Open Database License (ODbL) is a license agreement intended to allow users to freely share, modify, and use this Dataset while maintaining this same freedom for others, provided that the original source and author(s) are credited.Ivan Neo, Rebecca Ker Loh, Tricia J. Y. Cho, Nalini Puniamoorthy, Darren C. J. Yeo, Ming Kai Tan
